# Supplementary material for: Economical representation of spatial networks
Source: PNAS Nexus. 2025 Jun 18;4(7):pgaf203. doi: 10.1093/pnasnexus/pgaf203 (PMC12218191; doi:10.1093/pnasnexus/pgaf203)
Supplement: pgaf203_Supplementary_Data [file pgaf203_supplementary_data.pdf]

Supplementary Material

Economical representation of spatial networks

Fabrizio De Vico Fallani[1*], Thibault Rolland[1]

1 Sorbonne University, Paris Brain Institute (ICM), CNRS, Inria, Inserm, AP-HP, Pitie-Salpetriere Hospital, Paris, France

* corresponing author: fabrizio.de-vico-fallani@inria.fr

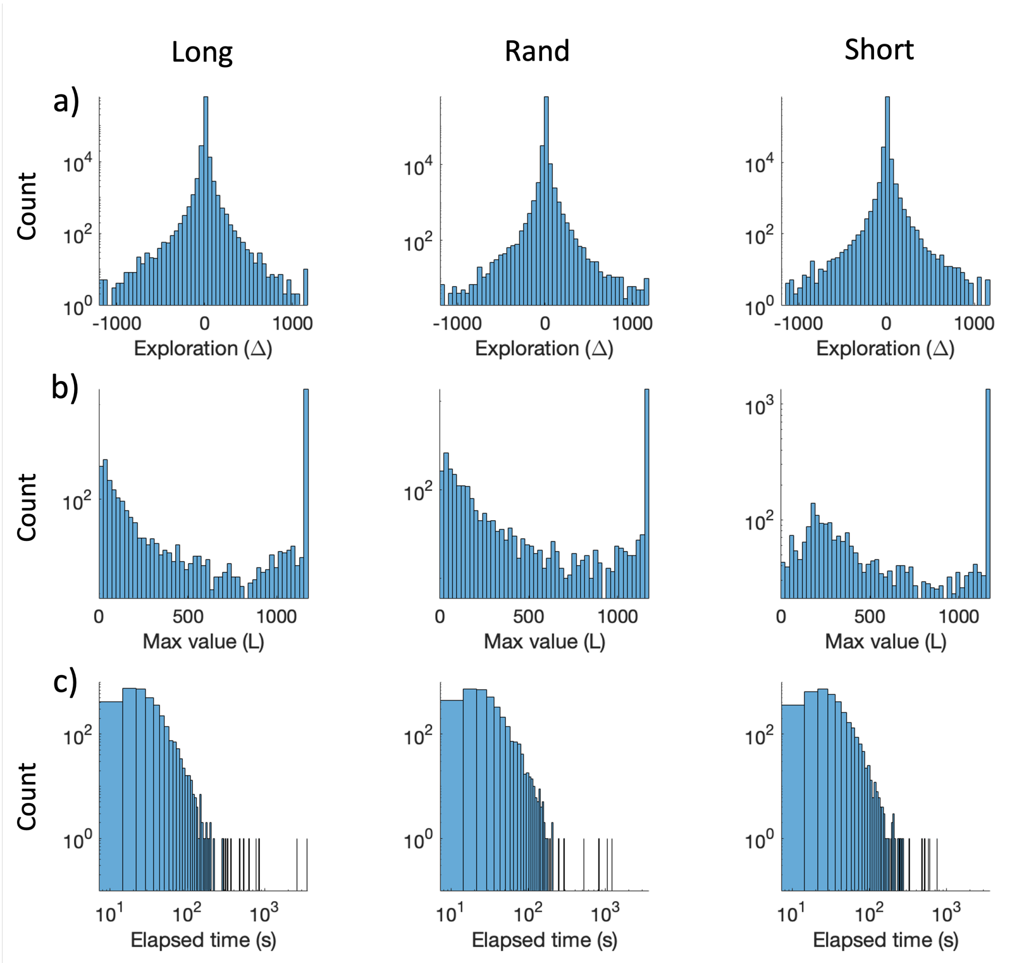

Fig S1. NetViz users' behavior. Distribution of the users' exploration trend (Δ) defined as the difference between two consecutive explored values L (number of visualized links). Positive/negative Δ indicate increment/decrement of the visualized links. For example, the negative value -1175 indicates an extreme jump from the maximal number of connections that can be visualized ($L = 1176$) to the minimum $L = 1$. Although the distributions look symmetric and centered on zero, the group-average indicates a global weak tendency to visualize an increasing number of links, i.e. $L^{long}_{max} = 1.1609$, $L^{rand}_{max} = 1.2754$, $L^{short}_{max} = 1.7829$. Notably, this mean tendency is not statistically different between conditions (Cohen's $|d| < 0.024$). b) Distribution of the maximum number of connections (L) visualized by the users. In every condition, most of the users visualized all the possible number of connections ($L = 1176$) before making their choice. Other users instead explored a relatively lower number of links before making their selection. The group means in the different conditions $L^{long}_{max} = 678.7753$, $L^{rand}_{max} = 648.7348$, $L^{short}_{max} = 723.3520$ are not statistically different (Cohen's $|d| < 0.16$). c) Distribution of the time elapsed before the users make the choice. Globally, users were relatively fast in making their choices (less than one minute). The group-averaged durations in each condition $long = 35.3045s$, $rand = 33.8082s$ and $short = 35.8518s$ are not statistically different (Cohen's $|d| < 0.06$).

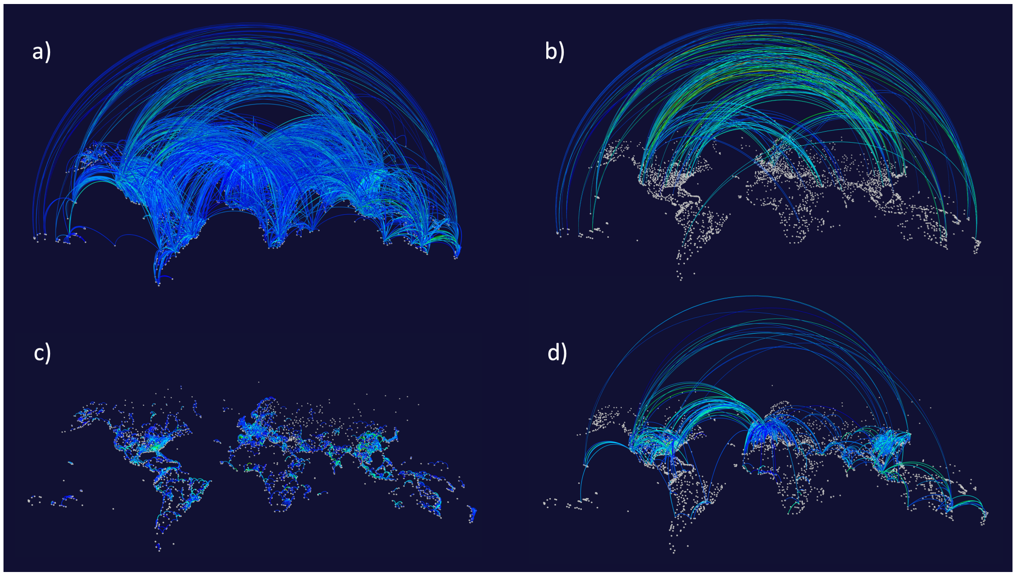

Fig S2. Orignal and filtered versions of the Airline route network. Nodes correspond to airports and links correspond to the number of operated flights. The link weight is coded by the color. The lighter the color, the higher the number of flights. For illustrative purposes, the network is shown on its 2D geographical representation. The height of the connections is proportional to the geodesic distance between the connected airports. a) Original network ($N = 3214$, $L = 18859$). b) Network filtered with $\phi = 1$ and $s = 3$. The optimal connection density is obtained by maximizing J and sorting the links by their decreasing length (long). Final number of filtered connections $L = 191$. c) Network filtered with $\phi = 1$ and $s = 3$. The optimal connection density is obtained by maximizing J and sorting the links by their increasing length (short). Final number of filtered connections $L = 4355$. d) Network filtered with $\phi = 1$ and $s = 3$. The optimal connection density is obtained by maximizing J and sorting the links by their actual weight. Final number of filtered connections $L = 915$. All visualizations are realized with the VIZAJ software [1]

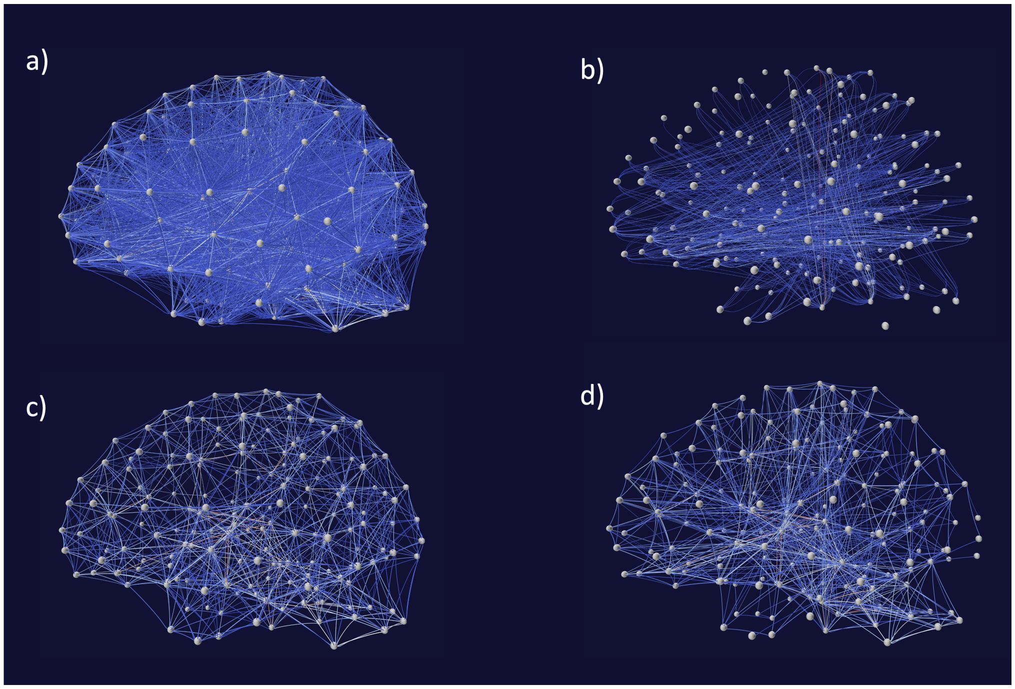

Fig S3. Orignal and filtered versions of the human connectome. Nodes correspond to different brain regions, and links measure the number of axonal fibers between different regions (in log scale). The link weight is coded by the color. The lighter the color, the higher the number of fibers connectivty the regions. a) Original network ($N = 188$, $L = 5446$). b) Network filtered with $\phi = 1$ and $s = 3$. The optimal connection density is obtained by maximizing J and sorting the links by their decreasing length (long). Final number of filtered connections $L = 468$. c) Network filtered with $\phi = 1$ and $s = 3$. The optimal connection density is obtained by maximizing J and sorting the links by their increasing length (short). Final number of filtered connections $L = 1327$. d) Network filtered with $\phi = 1$ and $s = 3$. The optimal connection density is obtained by maximizing J and sorting the links by their actual weight. Final number of filtered connections $L = 1077$. All visualizations are realized with the VIZAJ software [1]

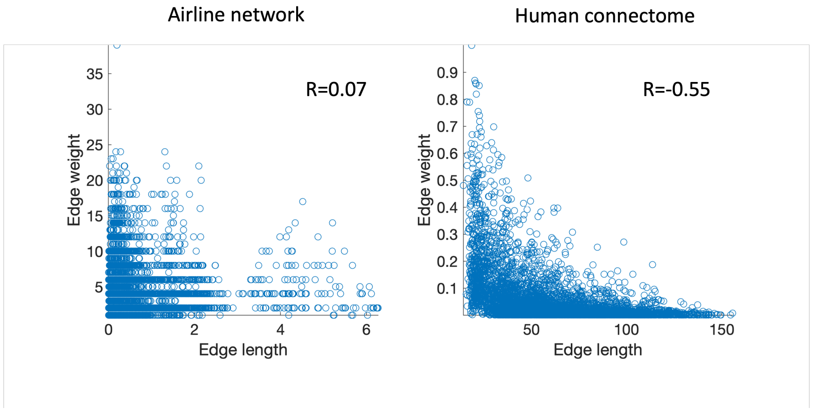

Fig S4. Rank correlation between the link weights and lengths of real networks. Scatter plots of the edge weights versus their length. Rank correlations are computed via the Spearman coefficient R, and show that only the human connectome exhibits a significant effect, indicating that brain areas that are spatially adjacent also tend to be more strongly connected.

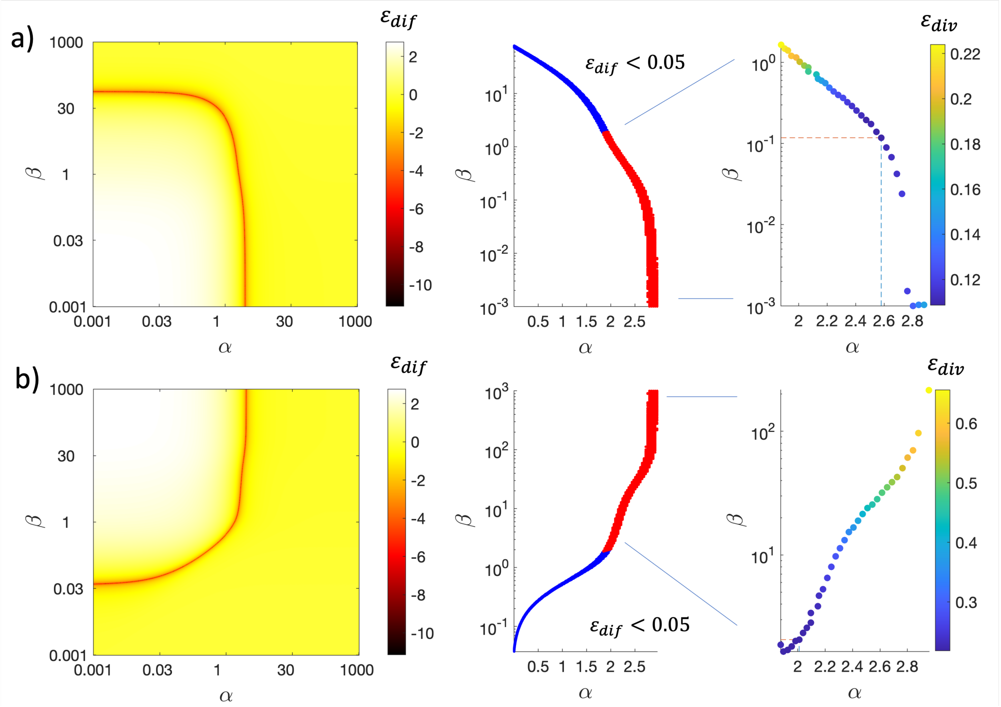

Fig S5. Model fitting results for the neuronal network. a) shows the results obtained when including the degree of the target node in the cost per length unit i.e. $c = k^{\alpha}\beta$ b) shows the results obtained when removing the degree of the target node from the cost per length unit, i.e. $c = \beta$. First column shows the values of the relative error in reproducing the actual number of links (ϵ_{dif}) over a wide parameter space spanning several order of magnitudes. Values are show in a logarithmic scale for the sake of readability. Second column highlights the parameter combination whose ϵ_{dif} values correspond to networks with $2287 \pm 5\%$ connections (red zones). The third column, shows the goodness-of-fit in terms of maximum Jensen-Shannon divergence (ϵ_{div}) obtained when comparing real and simulated networks in terms of node degree and edge length distribution (**Methods**). Dashed lines spot out the best global minumum $\alpha = 2.51$, $\beta = 0.18$.

1 Relations between connection and spatial density

Let us consider a weighted undirected geometric graph consisting of N nodes and L links, or edges, embedded in a s-dimensional space. For every edge l, the length $d_l \in [0,1]$ can be obtained by the Euclidean distance between its endpoints divided the maximal internode distance in the graph. Like the connection density, we can define the spatial density as

$$\delta = \frac{D_L}{D_{tot}} = \frac{\sum_{l=1}^{L} d_l}{\sum_{l=1}^{L_{tot}} d_l} \in [0,1] \tag{1}$$

which measures the proportion of distance covered by the L edges in the graph over the maximum when all the nodes are connected, i.e., $L_{tot} = N(N-1)/2$. Note that this definition does not take into account the edge weights. These are instead needed to establish analytic relationships between the connection and spatial density. Without loss generality, let us therefore assume that every edge l is also equipped with a positive weight $w_l \in [0,1]$.

Edge weights and lengths are not correlated

We first consider the scenario where a certain number L of links are assigned between the nodes in a completely random fashion. By construction, the edge weights and lengths are uncorrelated and selecting the links according to their weight will correspond to randomly select the lengths. Hence, assuming that all internode distances and link weights (are unique, the probability to select an edge with length d_l is equivalent to roll a fair die with L faces, i.e, $P(d_l) = 1/L$. The expected value $\langle d_l \rangle_{rand} = 1/2$ and substituting in Eq. 1 we obtain

$$\delta_{rand} = \frac{\sum_{l=1}^{L} \langle d_l \rangle_{rand}}{\sum_{l=1}^{L_{tot}} \langle d_l \rangle_{rand}} = \frac{L}{L_{tot}} = \rho \tag{2}$$

Edge weights and lengths are correlated

Next, we consider the scenario where the strongest links are assigned between the farthest (or closest) nodes. This procedure introduces a correlation between the weights and the lengths since the related rankings coincide. Now, selecting the links by their weight will correspond to select the distances in the same (or opposite) order. In this case, the probability to randomly pick an edge with length d_l is not uniform but depends on its position in the ranking.

Leveraging tools from basic distribution theory of order statistics, it is possible to estimate the expected value assuming that each distance is an independent random variable sampled from a common distribution $F(d_l)$. For the sake of simplicity, we first consider the case where F is absolutely continuous and uniform in the unitary interval.

If L_{tot} values are randomly sampled from the unitary interval and arranged in order, the l-th order statistics follows a $Beta(l, L_{tot} - l + 1)$ distribution, which is analytically tractable. Hence, when links are sorted in an increasing order starting from shortest distances, the expected value $\langle d_l \rangle_{short} = \frac{l}{L_{tot}+1}$. Conversely, if links are sorted in an decreasing order starting from longest distances, the expected value $\langle d_l \rangle_{long} = 1 - \langle d_l \rangle_{short} = \frac{L_{tot}-l+1}{L_{tot}+1}$. Substituting in Eq. 1 and using Gauss summation we obtain:

$$\delta_{short} = \frac{L}{L_{tot}}\left(\frac{L+1}{L_{tot}+1}\right) = \rho\frac{L+1}{L_{tot}+1} \simeq \rho^2$$
$$\delta_{long} = \frac{L}{L_{tot}}\left(\frac{2L_{tot}-L+1}{L_{tot}+1}\right) \simeq 2\rho - \rho^2 \qquad (3)$$

Note that, however, in general the typical internode distance is not uniform and the related k-th moments are not easy to evaluate analytically, as the shape of F cannot be reduced to a closed-form [2]. In particular, given a number of points arbitrarily positioned in the space there will be fewer long distances and the distance sum in Eq 1 will be in general smaller compared to an ideal uniform distribution (**Fig S6a**).

To have a general intuition on how the real case deviates from the ideal one, let us start with short-range configurations. If $P(d_l)$ is uniform, we have $D_L = \sum_{l=1}^{L}\langle d_l\rangle_{short} = \frac{L(L+1)}{2L_{tot}} \simeq \frac{L^2}{2L_{tot}}$. Note that L_{tot} is constant - given by the network size - and that the factor $L(L+1)/2 \simeq L^2/2$ is the sum of the first L integers corresponding to the ranks of the sorted links. If $P(d_l)$ is not uniform, distances might tend to accumulate around a central value like in the case of N points distributed on a plane (**Fig S6a**). The sum in terms of integers is not appropriate anymore because the related ranks do not correspond to the distribution of the real distances. To compensate this effect, the ranks should be real numbers so that the related L values can fit arbitrary distance distributions (**Fig S6b**).

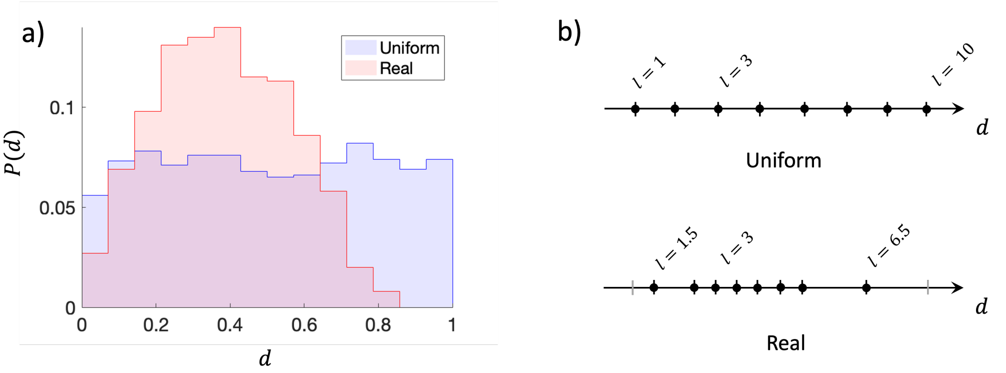

Fig S6. Effect of nonuniform internode distance distribution on the connection ranks. a) Distance distributions obtained by sampling 1000 values. Blue bars = values from a uniform distribution $U(0,1)$. Red bars = values randomly drawn from all the possible normalized distances between $N = 1000$ points arbitrarily positioned in the unitary square. The sum of the distances in the uniform case is higher than that in the real case, i.e. $D_L^{uni} = 505.65 > D_L^{real} = 383.27$. b) Schematic example of connection rank compression in the real scenario. Eight different ditances are considered here for the sake of simplicity. The distance distribution is properly represented by consecutive integer numbers in the uniform case. In the real one, the ranks can be real numbers so to better represent the actual nonuniform distance distribution. Accordingly, the sum of the ranks in the uniform case is higher than that in the real case, i.e. $55 > 30.5$.

Because the real distances do not span the entire interval $(0,1]$, but a reduced portion of it, the sum of the first L real ranks will be in general lower than the first L integers. Put differently, the sum of the ranks now scales with a power $\nu \leq 2$ of the number of links, i.e. $D_L = \sum_{l=1}^{L}\langle d_l\rangle_{short} \simeq \frac{L^\nu}{2L_{tot}}$. Substituting in Eq 1, the spatial density for short-range configurations in realistic scenarios reads

$$\delta_{short} \simeq \frac{L^{\nu}}{2L_{tot}} \frac{2L_{tot}}{L_{tot}^{\nu}} = \rho^{\nu} \tag{4}$$

Because $\langle d_l \rangle_{long} = 1 - \langle d_l \rangle_{short}$, we can derive after few steps the spatial density for long-range configurations in realistic scenarios:

$$\delta_{long} \simeq 2\rho \left(\frac{L_{tot}}{2L_{tot} - L_{tot}^{\nu-1}} \right) - \rho^{\nu} \left(\frac{L_{tot}^{\nu-1}}{2L_{tot} - L_{tot}^{\nu-1}} \right) \tag{5}$$

which tends exactly to $\delta_{long} = 2\rho - \rho^{\nu}$ as $\nu \to 2$.

Fig S7a shows that $\nu = 2$ nicely captures the behavior of real distances and provides the general limit boundaries. Adjusting ν to values slightly lower than two allows to be specific to each situation and obtain a perfect fit with the real data (**Fig S7b**). Importantly, the related connection density maximizing $J = \rho(1-\delta)$ does not significantly differ between the two options and get emphasized with Eqs 3. Indeed, using $\nu = 2$, one obtains a slightly higher number of links in short-range configurations and a slightly smaller amount in long-range configurations as compared to the actual results.

In conclusion, even if real distances typically breaks the uniformity assumption, Eqs 3 represent general solutions that can be used to derive a fair approximation of the analytical behavior.

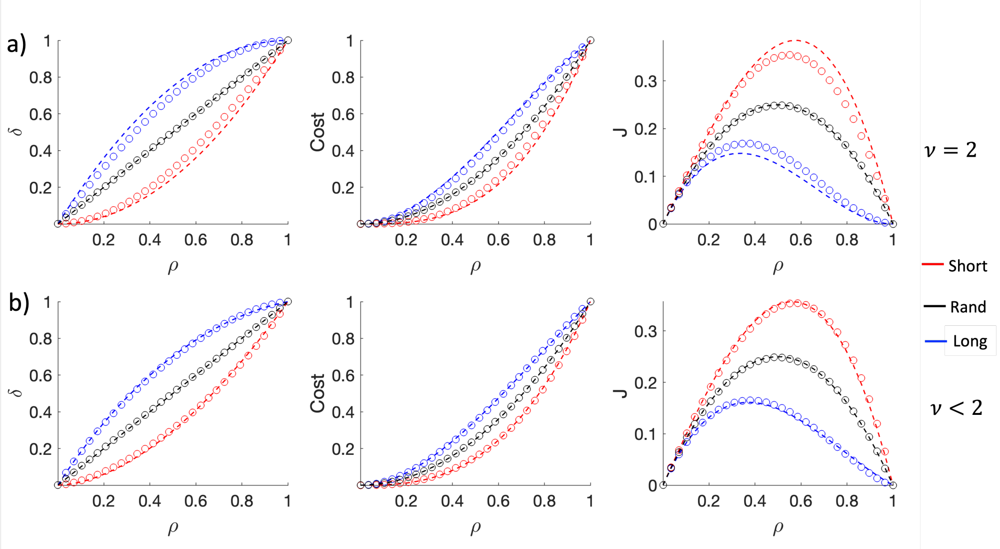

Fig S7. Theoretical versus empirical behavior of spatial density δ, cost and functional J. Dashed lines represent the values obtained from the analytical expressions. Circle markers represent the values obtained by simulating a synthetic network with $N = 1000$ nodes arranged randomly on a 2D unitary circle. For illustrative purposes, we have considered here the filtering parameter $\alpha = 1$, so that the cost $=\rho\delta$ and $J = \rho - \rho\delta$. a) Theoretical values obtained assuming a uniform distribution of the internode distances, i.e., $\nu = 2$ (Eqs 4-5). b) Theoretical values adjusting the exponent (ν) so as to fit the actual data coming from the simulated network, i.e., $\nu = 1.75$ (Eqs 4-5). Note that results stay qualitatively the same for any other sufficiently large number and arbitrary positioning of the nodes in the plane (data not shown here).

2 Analytical solution for optimal connection density

We now seek to find the optimal ρ maximizing the simplified functional $J = \rho^\alpha(1-\delta)^\beta$ with the controlling parameters $\beta = 1$ and $\alpha \geq 0$. In this case, the functional can be directly rewritten in terms of benefit-cost $J = \rho^\alpha - \rho^\alpha\delta$. By substituting the analytic expressions of the distances obtained in Eqs 2) and 3), the functional becomes a function of the connection density only

$$J = \begin{cases} \rho^\alpha - \rho^{\alpha+2}, & \text{if } \textit{short-range} \\ \rho^\alpha - \rho^{\alpha+1}, & \text{if } \textit{random} \\ \rho^\alpha - 2\rho^{\alpha+1} + \rho^{\alpha+2}, & \text{if } \textit{long-range} \end{cases} \tag{6}$$

By derivating with respect to ρ and equating to zero, we finally obtain a parametric solution for the optimal connection density in sufficiently big networks

$$\rho = \begin{cases} \sqrt{\dfrac{\alpha}{\alpha+2}}, & \text{if } \textit{short-range} \\ \dfrac{\alpha}{\alpha+1}, & \text{if } \textit{random} \\ \dfrac{\alpha}{\alpha+2}, & \text{if } \textit{long-range} \end{cases} \tag{7}$$

By letting the filtering parameter scale the typical internode distance $\alpha = \phi N^{-1/s}$ [3], where ϕ is a positive constant, we eventually obtain

$$\rho = \begin{cases} \sqrt{\dfrac{\phi}{\phi + 2N^{1/s}}} \simeq \sqrt{\dfrac{\phi}{2}} N^{-1/2s}, & \text{if } \textit{short-range} \\ \dfrac{\phi}{\phi + N^{1/s}} \simeq \phi N^{-1/s}, & \text{if } \textit{random} \\ \dfrac{\phi}{\phi + 2N^{1/s}} \simeq \dfrac{\phi}{2} N^{-1/s}, & \text{if } \textit{long-range} \end{cases} \tag{8}$$

where s is the dimension of the Euclidean space and the approximate equality holds for sufficiently large networks, i.e. $N \to \infty$.

Eqs 6 can be easily rewritten in terms of optimal number of links, i.e., $L_{long} \simeq \frac{\phi}{2} N^{2-1/s} < L_{rand} \simeq \phi N^{2-1/s} < L_{short} \simeq \sqrt{\frac{\phi}{2}} N^{2-1/2s}$. For Euclidean spaces with increasingly high dimensions (i.e., $s \to \infty$), the optimal number of edges scale exactly with the square of the network size.

In addition, for a given ϕ, high dimensional networks will contain more edges than low dimensional ones. This behavior is in line with the fact that the likelihood of having edge crossings in a 3D space ($s = 3$) is much lower than in 2D planes ($s = 2$).

3 Modeling the number of connections to display

The NetViz experiment asks users to select a number of edges connecting different points, or nodes, located on a pseudo-random 2D grid. This is equivalent to counting the number of events (i.e., the edges) that occur in a fixed space interval and can be modeled by a Poisson process. In a standard Poisson model the event occurrence rate is constant and the mean value equals its standard deviation.

Here, the occurrence rate is unknown and expected to vary across individuals because of the subjective nature of the experimental task. As a result the variance of the number of links L can be much larger than its mean λ. This behavior can be specifically captured allowing the parameter λ to vary as a Gamma distribution. The resulting Gamma-Poisson process can be then used to model the number of observed links

$$L \sim GP(\lambda, \gamma) = \frac{\Gamma(L + 1/\gamma)}{\Gamma(1/\gamma)\Gamma(L+1)}\left(\frac{\lambda^2\gamma}{\lambda + \lambda^2\gamma}\right)^L\left(\frac{1}{1+\lambda\gamma}\right)^{1/\gamma} \tag{9}$$

where Γ is the *gamma* function, λ is the expected mean value and the scale parameter γ accounts for the overdispertion of the data. Note that depending on the parametrization, the $GP(\lambda, \gamma)$ is equivalent to a negative binomial distribution $NB(r, p)$ where the number of trials $r = 1/\gamma$ and the number of successes $p = \dfrac{1}{1+\gamma\lambda}$ (**Fig S8**) [4].

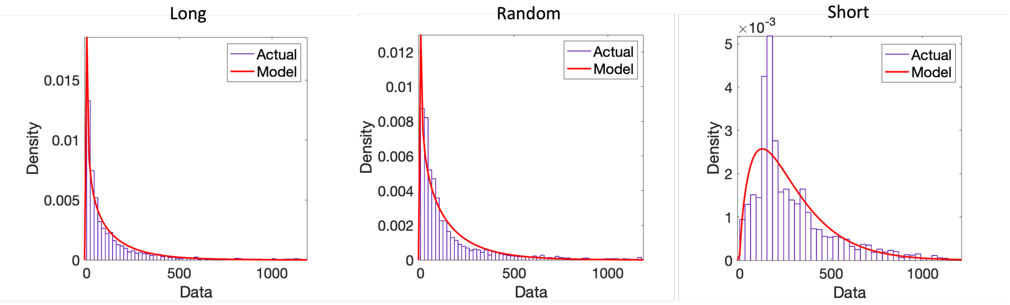

Fig S8. Number of links modeled by Gamma-Poisson processes. Model parameters calculated with maximum likelihood estimation (MLE) using Negative Binomial distributions $NB(r, p)$. a) Long-range. $r = 0.6844$ (std=0.01483), $p = 0.00518$ (std=0.0002). Mean=131.42, Variance=25366.4. b) Random. $r = 0.7751$ (std=0.01700), $p = 0.00515$ (std=0.0002). Mean=149.75, Variance=29080.7. c) Short-range. $r = 1.7933$ (std=0.0412), $p = 0.0063$ (std=0.0002). Mean=281.2, Variance=44373.5.

4 Relation between E_c and ρ in random graphs

We consider undirected geometric random graphs constituted by N nodes arbitrarily positioned in a 2D Euclidean space. Let p be the probability that two nodes are connected by a straight edge (i.e., a line segment). Edge crossing occurs when two lines intersect nontrivially, i.e., excluding partial and complete node coincidences. In a 2D plane this can only occurs when there are at least 4 nodes. Similar to [5], one can therefore estimate the number of times that any $k = 2$ pairs of nodes are connected in all subgraphs of size $m = 4$, i.e., $\binom{N}{m=4}p^{k=2}$.

In complete graphs ($p = 1$), this quantity corresponds to the maximal number of edge crossings E_c^{max} only if every subgraph gives an intersection. A necessary condition for this to happen is that every group of four nodes form a convex quadrilateral. It is trivial to show that this condition is respected when the N nodes are located on a circle. More in general, only a portion of all the possible subgraphs will lead to an intersection and $E_c^{max} = C\binom{N}{4} \leq \binom{N}{4}$, where C is the probability that the four related nodes form a convex quadrilateral (**Fig S9a**). Previous studies demonstrated that C is bounded between 2/3 and 1 depending on the specific geometry of the nodes. For example, $C = 1$ when nodes lie on a circle. $C = 2/3$ when nodes are within a triangle while $C = 25/36$ when nodes fall within a square area [6].

Because edges are independent in random graphs, C does not vary with the connection probability p and the actual number of edge crossings is (**Fig S9b**)

$$E_c = E_c^{max}p^2 = C\binom{N}{4}p^2 \tag{10}$$

Note that for a given p the expected number of links $L = p\frac{N(N-1)}{2}$ and the related connection density $\rho = p$. Substituting in Eq 10, we obtain the number of edge crossings as a function of the number of links

$$E_c = C\frac{(N-2)(N-3)}{6N(N-1)}L^2 \approx \frac{C}{6}L^2 \tag{11}$$

Taken together, Eqs. 10-11 extend previous results obtained for fully connected graphs and indicate that E_c scales with the square of the number of existing edges in a geometric random graph. Apart from the above mentioned theoretical cases, the precise values can be obtained by calculating the constant C for example by counting the number of convex subgraphs of four nodes over the total possible ones.

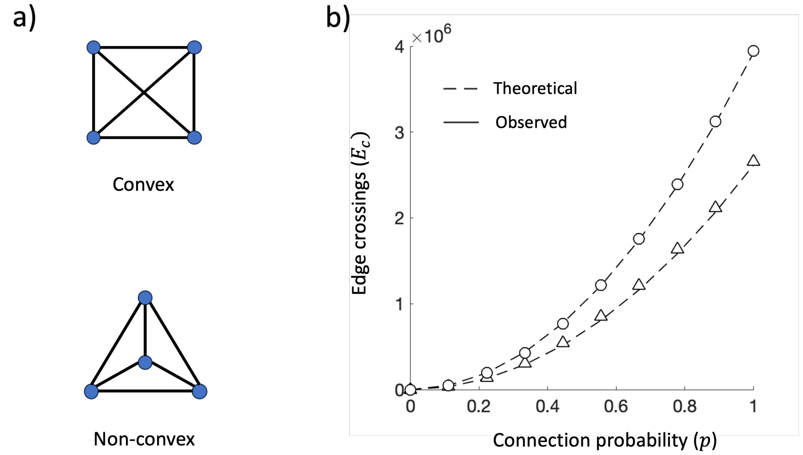

Fig S9. Edge crossings dependency on the network geometry and density. a) shows two examples of complete subgraphs with $m = 4$ nodes. The top one forms a convex quadrilateral and necessarily leads to an intersection. The bottom one is not convex and does not give intersections. b) shows the number of actual edge crossings E_c in random graphs as a function of the connection probability p. Solid markers correspond to values calculated with the Bentley-Ottman algorithm from an instance of a simulated graph with $N = 100$ nodes. Dashed lines correspond to theoretical values (Eq 10). Circles=nodes are spatially arranged on a circle ($C = 1$). Triangles= nodes are spatially arranged within a triangle ($C = 2/3$).

5 NetViz development and user profiles

5.1 Software implementation and survey

NetViz does not need to be installed on the personal computer. Everything works on a front-end browser and on a dedicated back-end server hosted by the Amazon Web Service. NetViz has been implemented in Python (back-end) and TypeScript (front-end). The graphs are generated back-end and displayed with Plotly on the front-end via the REACT javascript framework. Data are stored using the database storage SQLITE on the back-end. To adapt to possibly different displays the position of the nodes linearly scales with the window height, while nodes keep their sizes. The edge width is fixed to 1 pixel when the window's height is lower than 15 inches and 2 pixels for larger heights.

In the following we provide all the questions of the survey in the order they appear:

- **Page 1 (informative)**. We study the best way to represent complex networks. The Netviz project aims to measure how individuals perceive complex diagrams. To this end, we implemented a simple game that allows you to interactively connect different points of a graph. Your task? Choosing the number of connections to visualize - Play the Game - There's no right or wrong answer, you decide - Do you think the graph is comprehensible by showing more or less connections? Maybe it's somewhere in the middle? Make your choice. The game gives players the freedom to explore and choose the best number of connections based on their personal taste and opinion - 10 seconds of your time, a big step for our study We aim to collect a very large number of answers to quantify how people perceive complex networks and to understand if there is an optimal way to visualize their connectivity. The game is completely anonymous and does not save any type of personal data.

- **Page 2 (the survey)**. Use the slider to keep adding connections until the graph becomes too confusing. The text in the entries may be of any length - Press OK

to validate your choice.

5.2 User recruitment and demographics

NetViz has been translated in eight different languages to ease its dissemination across the world, i.e., French, Italian, English, Spanish, Portuguese, Arab, Chinese, Russian. Users had the possibility to share the NetViz link with their contacts after they made their choice. The first batch of 500 users have been recruited via local means, i.e. mailing lists, social media (Linkedin, X), printed flyers and personal contacts. The second batch of $n = 10187$ user were recuited via the Prolific platform, specialized for gathering reliable responses to surveys from human beings (`prolific.com`). Overall, we collected answers from 58 different countries (**Fig S10**). Notably, the result trends were the same between the the first and second batch (**Fig S11**).

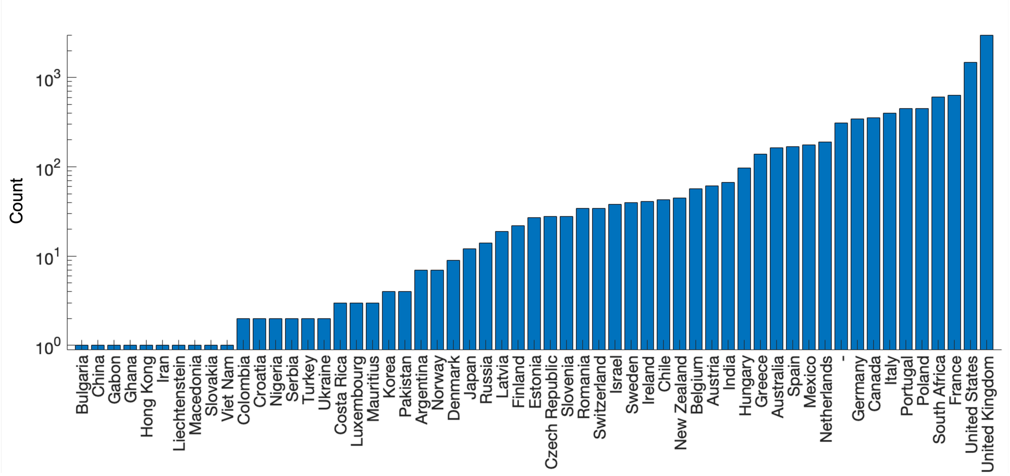

Fig S10. NetViz demographic distribution of the users by country. In general, most of the users participated from developed countries. The '-' symbol indicates thes users for which it was not possible to identify the country from the IP. Counts are in logarithmic scale.

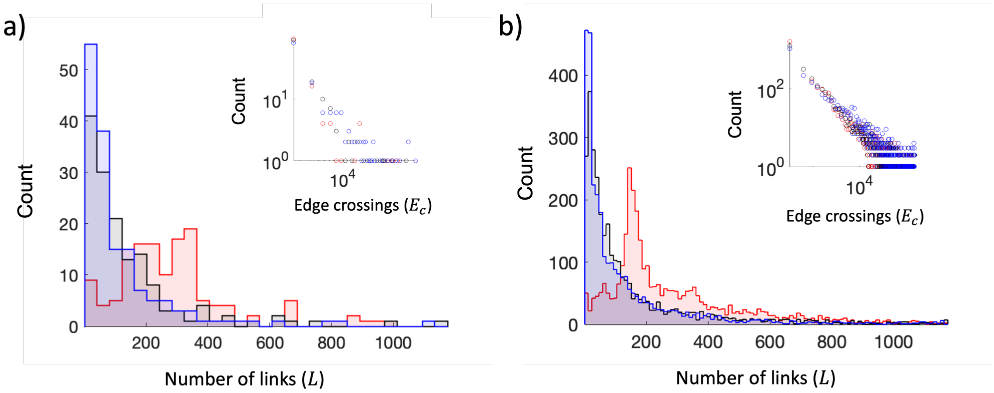

Fig S11. Analysis of the results based on the Local and Prolific dataset. a) The first set of data consists of $n = 500$ choices made by users contacted via local means, i.e. mailing lists, social medias, printed flyers and personal contacts. Results are qualitatively and quantitatively similar to those obtained considering the aggregated dataset (Local+Prolific, **Fig 2b** main text). In the short-range condition the average number of links ($L_{short} = 293.49$) is statistically higher than in the random ($L_{rand} = 142.07$, Cohen's $d = 0.8048$) and long-range condition ($L_{long} = 116.61$, Cohen's $d = 0.9872$). No statistical differences between L_{rand} and L_{long} (Cohen's $d = 0.1497$) nor between the estimated edge crossings (Cohen's $|d| < 0.255$).
b) The second dataset consists of $n = 10187$ choices made by users contacted via the Prolific platform (`prolific.com`). Results are qualitatively and quantitatively similar to those obtained considering the aggregated dataset (Local+Prolific, **Fig 2b** main text). In the short-range condition the average number of links ($L_{short} = 280.66$) is statistically higher than in the random ($L_{rand} = 150.13$, Cohen's $d = 0.6203$) and long-range condition ($L_{long} = 132.18$, Cohen's $d = 0.7209$). No statistical differences between L_{rand} and L_{long} (Cohen's $d = 0.0901$) nor between the estimated edge crossings (Cohen's $|d| < 0.3$).

References

1. Rolland T, De Vico Fallani F. Vizaj—A free online interactive software for visualizing spatial networks. PLOS ONE. 2023;18(3):e0282181. doi:10.1371/journal.pone.0282181.
2. Arnold BC, Balakrishnan N, Nagaraja HN. A First Course in Order Statistics. SIAM; 2008.
3. Moltchanov D. Distance distributions in random networks. Ad Hoc Networks. 2012;10(6):1146–1166. doi:10.1016/j.adhoc.2012.02.005.
4. Hilbe JM. Modeling Count Data. Cambridge: Cambridge University Press; 2014. Available from: `https://www.cambridge.org/core/books/modeling-count-data/BFEB3985905CA70523D9F98DA8E64D08`.
5. Bollobas B, Erdös P. Cliques in random graphs. Mathematical Proceedings of the Cambridge Philosophical Society. 1976;80(3):419–427. doi:10.1017/S0305004100053056.
6. Scheinerman ER, Wilf HS. The Rectilinear Crossing Number of a Complete Graph and Sylvester's "Four Point Problem" of Geometric Probability. The American Mathematical Monthly. 1994;101(10):939–943. doi:10.2307/2975158.
